# Extracts of *Euphorbia nivulia* Buch.-Ham. showed both phytotoxic and insecticidal capacities against *Lemna minor* L. and *Oxycarenus hyalinipennis* Costa

**DOI:** 10.1371/journal.pone.0250118

**Published:** 2021-04-30

**Authors:** Muhammad Younus, Muhammad Mohtasheemul Hasan, Sajjad Ali, Bushra Saddq, Gulam Sarwar, Muhammad Irfan Ullah, Ambreen Maqsood, Sunny Ahmar, Freddy Mora-Poblete, Farazia Hassan, Jen-Tsung Chen, Ahmed Noureldeen, Hadeer Darwish

**Affiliations:** 1 Faculty of Pharmacy, Department of Pharmacognosy, The Islamia University of Bahawalpur, Bahawalpur, Pakistan; 2 Faculty of Pharmacy & Pharmaceutical Sciences, Department of Pharmacognosy, University of Karachi, Karachi, Pakistan; 3 Department of Entomology, UCA & ES, The Islamia University of Bahawalpur, Bahawalpur, Pakistan; 4 Faculty of Sciences, Department of Botany, The Islamia University of Bahawalpur, Bahawalpur, Pakistan; 5 Department of Entomology, College of Agriculture, University of Sargodha, Sargodha, Pakistan; 6 Department of Plant Pathology, The Islamia University of Bahawalpur, Bahawalpur, Pakistan; 7 Institute of Biological Sciences, University of Talca, Talca, Chile; 8 Department of Biotechonolgy and Bioinformatics, Virtual university of Pakistan, Bahawalpur, Pakistan; 9 Department of Life Sciences, National University of Kaohsiung, Kaohsiung, Taiwan; 10 Department of Biology, College of Sciences, Taif University, Taif, Saudi Arabia; 11 Department of Biotechnology, College of Sciences, Taif University, Taif, Saudi Arabia; Universite d’Orleans, FRANCE

## Abstract

Many phytochemicals can affect the growth and development of plants and insects which can be used as biological control agents. In this study, different concentrations of crude, hexane, chloroform, butanol, and aqueous extracts of *Euphorbia nivulia* Buch.-Ham., an endemic plant of the Cholistan desert in South Punjab of Pakistan, were analysed for their chemical constituents. Their various concentrations were also tested for their phytotoxic and insecticidal potential against duckweed, *Lemna minor* L., and the dusky cotton bug, *Oxycarenus hyalinipennis* Costa. various polyphenols, i.e., quercetin, gallic acid, caffeic acid, syringic acid, coumaric acid, ferulic acid, and cinnamic acid were detected in different concentrations with different solvents during the phytochemical screening of *E*. *nivulia*. In the phytotoxicity test, except for 100 μg/mL of the butanol extract gave 4.5% growth regulation, no phytotoxic lethality could be found at 10 and 100 μg/mL of all the extracts. The highest concentration, 1000 μg/mL, of the chloroform, crude, and butanol extracts showed 100, 63.1, and 27.1% of growth inhibition in duckweed, respectively. In the insecticidal bioassay, the highest *O*. *hyalinipennis* mortalities (87 and 75%) were recorded at 15% concentration of the chloroform and butanol extracts of *E*. *nivulia*. In contrast, the lower concentrations of the *E*. *nivulia* extracts caused the lower mortalities. Altogether, these findings revealed that *E*. *nivulia* chloroform extracts showed significant phytotoxicity while all the extracts showed insecticidal potential. This potential can be, further, refined to be developed for bio-control agents.

## 1. Introduction

In nature, living organisms compete for food, space, water, and light within their communities. Plants exercise chemical interactions with other plants and biotic components around their vicinity by discharging various secondary metabolites called allelochemicals [1]. These allelochemicals may have positive or negative impacts on other plants and animals. Plants produce a vast array of phytochemicals to ensure plant survival in the stresses caused by hostile plants, animals, insects, and microbes. These phytochemicals can crumble the competing plants by affecting their growth or causing chlorosis and wilting, leading towards the death of hostile plants termed phytotoxicity [1,2].

Many plants, including weeds, have been investigated for their phytotoxic potential against different weeds [3]. These plants inhibit the germination and growth of different crops by releasing certain water-soluble phytotoxins into their adjacent environment [4,5]. Alien species chemicals are allelopathic to the native plant species that invade plants to establish in a new ecosystem and environment [6]. Most of the active allelochemicals include cinnamic acids, flavonoids, and various terpenes are considered good sources of natural herbicides [7]. In addition to have phytotoxicity, allelochemicals are also found to be involved in plant-insect interactions with their insecticidal capacities [8]. In agricultural practices, the insecticidal capacity of plants can be used to manage insect pests, which are a serious problem by decreasing the yield of crops [3].

In agriculture and forest management, weeds and insect pests cause substantial obstructions in the growth and productivity of crops and trees. Hence, tons of pesticides are applied to the agrosystem for better outcomes [9]. Chemical pesticides for crop protection cause environmental contamination due to toxic residues accumulation in addition to pesticide resistance development in pest organisms [10]. For sustainable agriculture, there is a demand to screen potential phytochemicals for phytotoxic and entomotoxic capabilities to strengthen the integrated pest management system [11]. For such purpose, several natural plant compounds were identified as active ingredients of many pesticides. It has been reported that extracts of *Azadirachta indica*, *Calotropis procera*, *Cassia fistula*, *Chrysanthemum coronarium*, *Lantana camara*, *Murraya koenigii*, and *Punica granatum* showed insecticidal or nematocidal effects against some insects with high safety-index to mammals. Therefore, phytochemicals would be promising bio-control agents in sustainable agriculture in the future [12].

Among angiosperms, Euphorbiaceae family is comprised of about 340 genera and about 8000 species [13]. It has been reported that many phytochemicals of Euphorbiaceae plants showed insecticidal, larvicidal, ovicidal actions against different insects [14,15]. *Euphorbia tirucalli*, *E*. *pulcherrima*, and *E*. *antiquorum* are known to have good larvicidal properties against mosquitoes and other insects [16]. Additionally, *E*. *pulcherima* extracts also exhibited insecticidal activity against fall armyworm with toxic effect and higher insect growth regulation (IGR) activity [17].

*Euphorbia nivulia* Buch.-Ham. has gained the researcher’s attention due to its outstanding biological activities, but there is still limited information reported [18]. Northern and central India is the native habitat of this plant, and it is also found in Myanmar and Pakistan [13]. It contains compounds of diterpenes and triterpenes [19]. The latex contains alkaloids, glycosides, phenolic compounds, tannins, and terpenes [18]. In a previous study, the aqueous leaf extract revealed good toxicity and insect growth regulation (IGR) against *Plutella xylostella* [20].

*Oxycarenus hyalinipennis* Costa, commonly known as dusky cotton bug, is an emerging insect pest of cotton. Generally, it causes severe damage at the seedling stage. Adults of dusky cotton bugs are 4–4.3 mm long, having black thorax with shining white wings, while nymphs are pinkish with red-orange abdomen [21]. Their heavy infestation causes multiple injuries resulted into reduced seed oil, seed weight, and cotton yield. They inject toxic saliva into bolls leading towards greasy spots appearance [22]. It has been reported that this widespread species can also be found in Southern Europe [23]. There were previous studies that investigated the utilization of plant chemicals to suppress this pest [12,24].

The present study aimed to test the phytotoxic and insecticidal capacity of *E*. *nivulia* extracts against *Lemna minor* and *O*. *hyalinipennis*, respectively, to reveal its biological control potentials.

## 2. Material and methods

### 2.1. Preparation of plant extracts

The aerial parts (leave, stems and flowers) of *E*. *nivulia* were collected from desert areas (29.3892848° N, 71.7878353° E) of Bahawalpur, Pakistan in a local field farm of the university and do not need a permit. The plants were identified and authenticated by the taxonomist of the Department of Botany, The Islamia University of Bahawalpur, Pakistan with voucher specimen no. EN-AP-05-12-041. The collected plant material was segmented into pieces and dried under shade at room temperature for 40 days. Later, it was ground into powder using an electric grinder and sieved through a mesh (No. 60). Then, 100 g plant powder was dissolved in 1000 ml of ethanol (70%) at room temperature for 15 days with stirring (once a day). Then, the mixture was filtered three times with muslin cloth separately. Further, filtration was performed by filter paper (Whatman Grade-1). This filtrate was then evaporated under low pressure (-760mm Hg) and temperature on a rotary evaporator (Heidholph Laborota, 4000-efficient, Germany). It resulted in a thick semi-solid brownish gummy mass that was placed in the oven for drying (Memmert Beschichung Loading, Model 100–800, Germany). Then, dried material was weighed, labeled, and stored at 4°C after the percent yield calculation for further experimentation. The same procedure was repeated using other solvents (hexane, chloroform, butanol, water).

### 2.2. Phytochemical screening

To identify different phytoconstituents (alkaloids, flavonoids, glycosides, phenols, saponins, tannins, etc.), qualitative phytochemical screening of the plant crude extract and its different solvent-based fractions was performed using standard procedures described by World Health Organization (WHO)‎. Total phenolic content (TPC) was quantified, using Folin-Ciocalteu’s technique, as mg gallic acid equivalent (GAE) per gram of the extract [25]. Total flavonoid content (TFC) was assessed by the modified colorimetric method [26]. High-Performance Liquid Chromatography (HPLC) of the extracts was also performed at Central Hi-Tech Laboratory, University of Agriculture, Faisalabad, Pakistan as described previously by Pak-Dek et al., in 2020. The test samples were hydrolyzed by mixing and homogenizing 50 mg of plant extracts in 24 mL methanol. Then, 16 ml of distilled water was added followed by 10 ml of HCl (6M). This mixture was, then, heated for 2h at 95°C. The resultant solution was filtered through a 0.45 μm nylon filter before HPLC analysis. The HPLC analysis was carried out using Waters HPLC system furnished with Waters-2487 dual wavelength absorbance detector, Waters-600 Pump and controlled by Waters empower 2-software (Waters, Milford, MA). The separations were done using Waters reverse-phase (RP) Symmetry C-18 column (150*3.9 mm, 5 μm) at room temperature. The mobile phase comprised of de-ionized water with TFA (pH 2.5) as solvent-A and 99.99% methanol as solvent-B. The following gradient was used: 100–50% solvent-A (0 to 20 min), 50–40% solvent-A (20 to 30 min) and 40–100% solvent-A (30 to 40 min). The mobile-phase flow rate was held at 1 ml/ min and the detector was kept at 280 nm. Identification of phenolic compounds was established by comparing the retention time and UV-Visible spectra of the peaks with those previously obtained by injection of standards. Results were compared with the internal library of analytes maintained at Hi-Tech Lab. Quantification was performed by external calibration. The peak identifications and quantifications were done based on the judgment of retention time and area of standards, correspondingly [27].

### 2.3. *Lemna minor* culture and phytotoxicity bioassay

*L*. *minor* plants with 2–3 fronds were collected from the local university pond for this experiment. The pond water was filtered and autoclaved to use in growth media for *L*. *minor*. A phytotoxic bioassay was performed using an E-medium, prepared by mixing 0.68 g KH_2_PO_4_, 1.5 g KNO_3_, 1.18 g Ca(NO_2_)_2_.4H_2_O, 0.49 g 0.0028 g H_3_BO_3_, 0.0036 g MnCl_2_.4H_2_O, 0.0054 g FeCl_2_.4H_2_O, 0.0002 g ZnSO_4_.5H_2_O, 0.0002 g CuSO_4_.5H_2_O, 0.00012 g Na_2_MO_4_.2H_2_O and 0.0112 g EDTA in 1000 ml distilled water and adjusting pH between 6–7 by adding KOH (Stock solution). 100 ml stock solution was added to 900 ml distilled water to make a working E-medium. Similarly, 30 mg crude extract was also dissolved in 1.5 ml Ethanol solvent serving as stock solute. Three flasks were inoculated with 10, 100, and 1000 μl of solution from the stock solution for 10, 100 and 1000μg/ml. 20 ml working E-medium and *L*. *minor* plants (with a rosette of two to three fronds) were added to each flask (a total of 20 fronds). Other flasks were supplemented with E-medium and reference (standard-drug) plant growth inhibitors and promoters as -ve and +ve control treatments. These flasks were positioned in growth chambers for seven days. The treated plants were observed daily for plant growth, and the number of fronds in each flask was recorded on day 7. Results were analysed as percent growth regulation regarding -ve control using the following equation:

$$Percent\;regulation=100-\frac{No.\;of\;fronds\;in\;test}{No.\;of\;fronds\;in–ve\;control}*100$$

Following criterion was used for assessing phytotoxicity: 0–39% growth inhibition was low activity, 40–59% inhibition was moderate activity, 60–69% inhibition was an excellent activity, and above 70% inhibition was significant activity [28].

### 2.4. *Oxycarenus hyalinipennis* culture and insecticidal bioassay

*O*. *hyalinipennis* adults were collected from university research fields (The Islamia University of Bahawalpur, Pakistan) during 2019 and reared under laboratory conditions (25±2°C, and 65±5% R.H.). The insect population was maintained on their natural food (water-soaked fuzzy seeds of cotton) in plastic chambers (36×60×60 cm) provided with the aerial flow till next generation and third instar nymphs from this generation were used in experiments. Twenty *O*. *hyalinipennis* nymphs (3^rd^ instar), to have a uniform population, were positioned in Petri-dish (15 cm diameter) containing cotton seedlings with roots enclosed by wet cotton to keep them alive. Afterwards, they were sprayed with 5, 10, and 15% solutions of crude, aqueous, butanol, chloroform, and hexane extracts of *E*. *nivulia* and insecticide Oberon (spiromesifen), using a fine hand-sprayer machine (Flip & Spray™ Bottles, Thomas Scientific, USA). A control treatment was maintained by applying water spray. The treatments were triplicated under laboratory conditions (25±1°C, 65±5% RH) [29].

### 2.5. Data analysis

Evaluation of the toxicity of different treatments was based on the corrected mortality percentage calculated through Abbot’s formula [30]:

$$Coreected\;\%\;mortality=\left(1-\frac{\;n\;in\;T\;after\;treatment}{n\;in\;Co\;after\;treatment}\right)*100$$

Where n = insect population, T = treated, Co = control.

The corrected percent mortality data were analysed using factorial analysis of variance (ANOVA) by Minitab 16.1 software application. The means were separated via Tukey’s HSD test at a 5% significance level to quantify the treatment’s impact.

## 3. Results

### 3.1. Phytochemical screening

The *E*. *nivulia* extracts contain many phytochemicals like glycosides, alkaloids, saponins, flavonoids, phenols, tannins, carbohydrates, and their types were highly affected by the extracting solvents (Table 1) [31,32].

**Table 1:** Phytochemical evaluation of various extracts of *Euphorbia nivulia* Buch.-Ham.

Test	Crude Aq. EtOH Extract	Hexane Extract	Chloroform Extract	Butanol Extract	Aqueous Extract
**Carbohydrates**					
*Fehling’s Test*	+	-	+	+	+
**Alkaloids**					
*Hager’s Test*	+	-	-	-	-
*Mayer’s Test*	+	-	-	-	-
*Wagner’s Test*	+	-	-	-	-
**Glycosides**					
*Keller Kiliani Test*	+	-	-	-	-
**Phenolic Compounds**					
*FeCl*_*3*_ *Test*	+	-	+	+	+
**Flavonoids**					
*Alkali Test*	+	-	+	+	+
**Tannins**					
*FeCl*_*3*_ *Test*	+	-	+	+	+
**Saponins**					
*Froth Test*	+	-	-	+	+

### 3.2. Total flavonoid and phenolic content (TFC and TPC)

**Fig 1:**
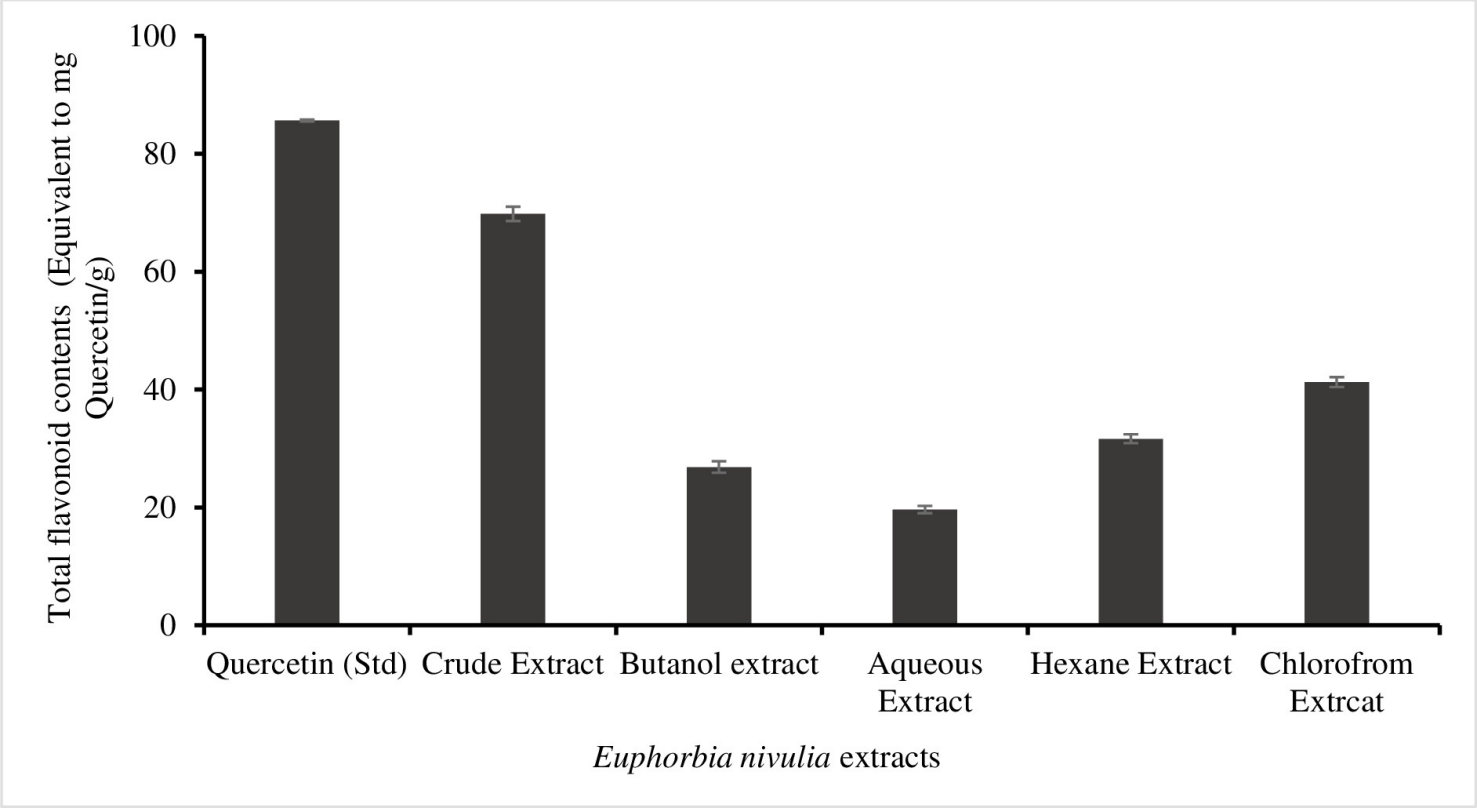
Total flavonoid contents in *Euphorbia nivulia* Buch.-Ham. crude extract and various fractions.

**Fig 2:**
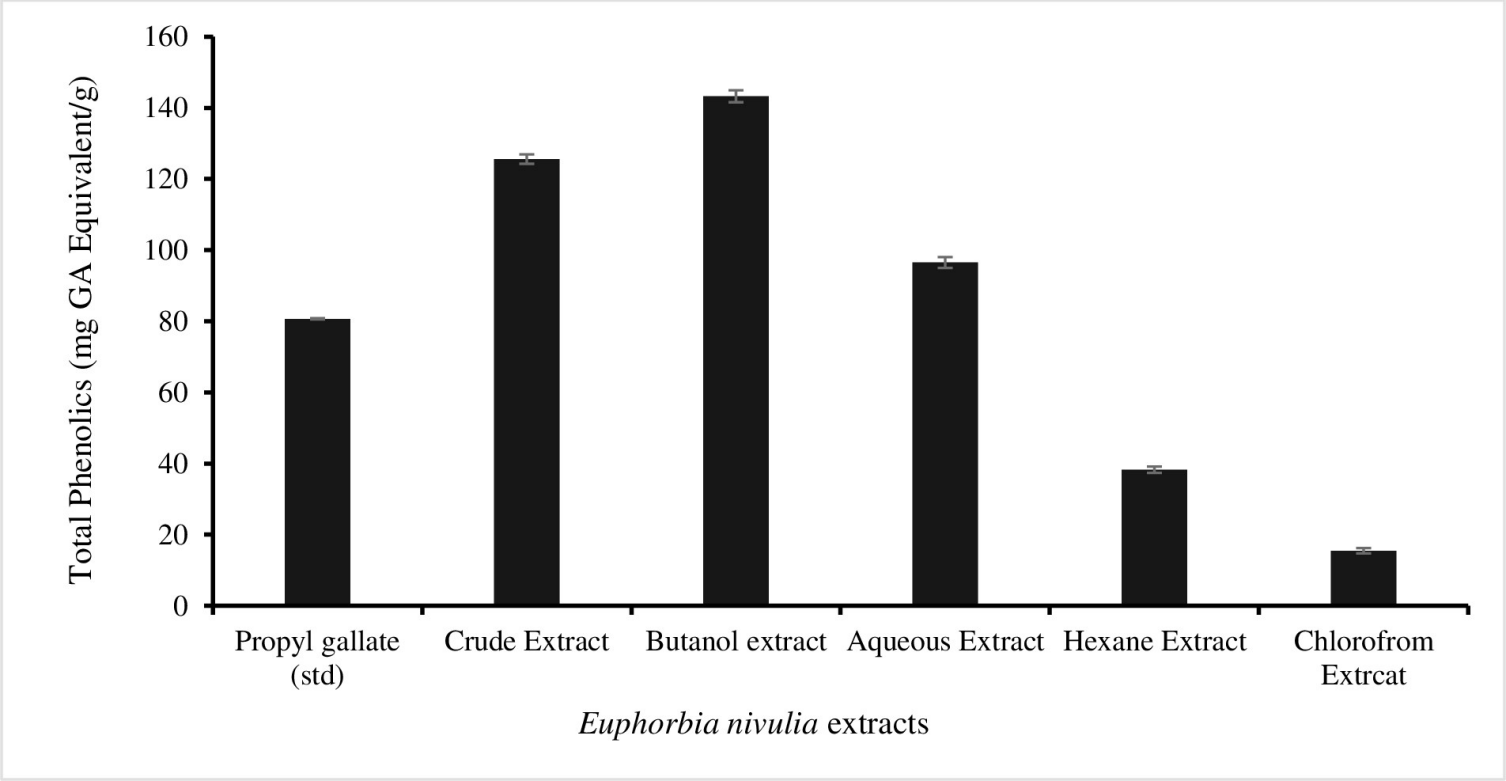
Total phenolic contents in *Euphorbia nivulia* Buch.-Ham. crude extract and various fractions.

The highest flavonoid content of 69.80±1.21 mg/g was found in the *E*. *nivulia* crude extract (Fig 1). Followed by 41.26±1.23, 31.65±1.22, 26.85±0.93, and 19.63±1.14 mg/g were found in crude, hexane, butanol and aqueous extracts, respectively. In the analysis of total phenolic contents, the highest was 143.26±2.65 mg/g in the butanol extract, followed by the crude extract of 125.6±1.32 mg/g, the aqueous extracts of 96.53±2.01 mg/g, the chloroform extract of 38.27±3.21 mg/g, and the hexane extract of 15.49±1.92 mg/g (Fig 2).

### 3.3. HPLC analysis

**Fig 3:**
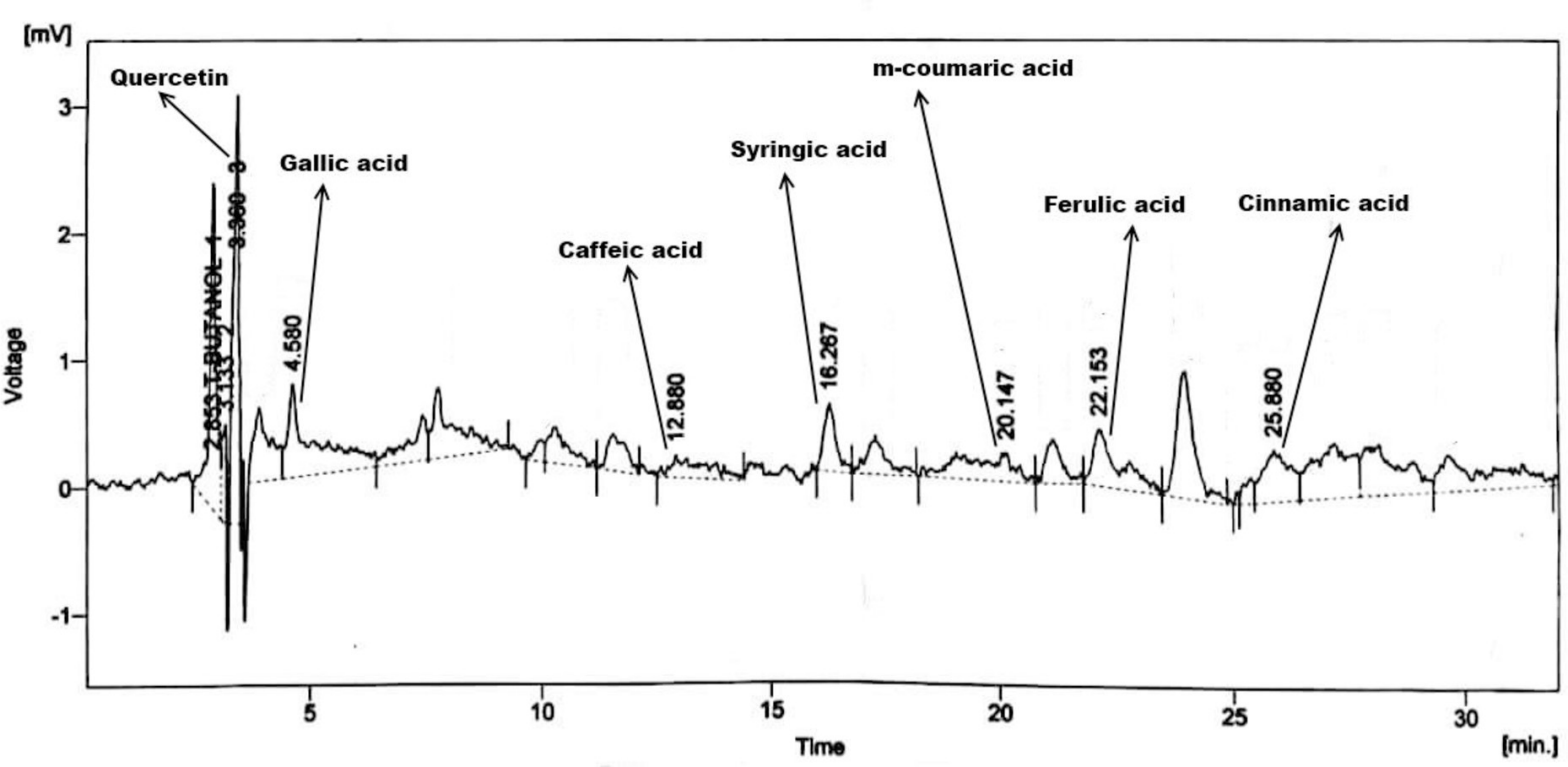
HPLC chromatogram of *Euphorbia nivulia* Buch.-Ham. crude extract.

**Fig 4:**
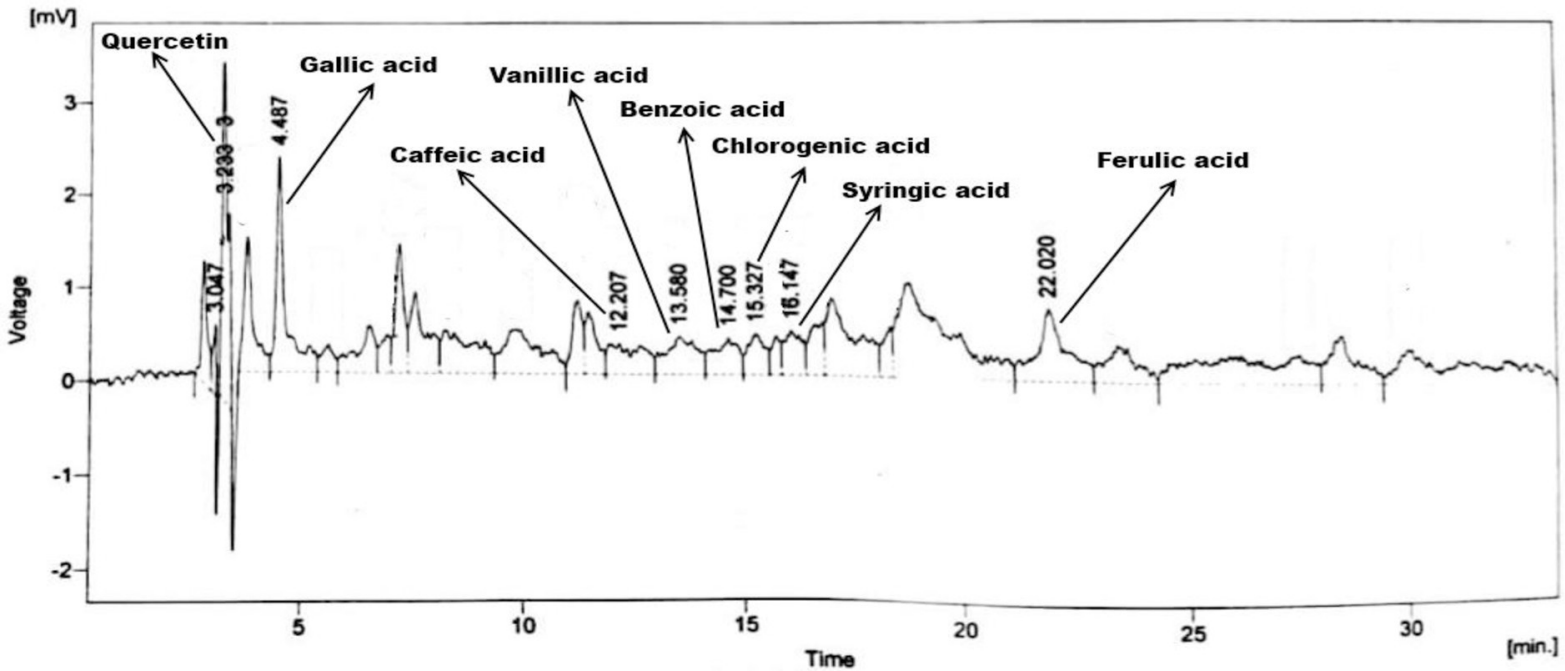
HPLC chromatogram of *Euphorbia nivulia* Buch.-Ham. aqueous extract.

**Fig 5:**
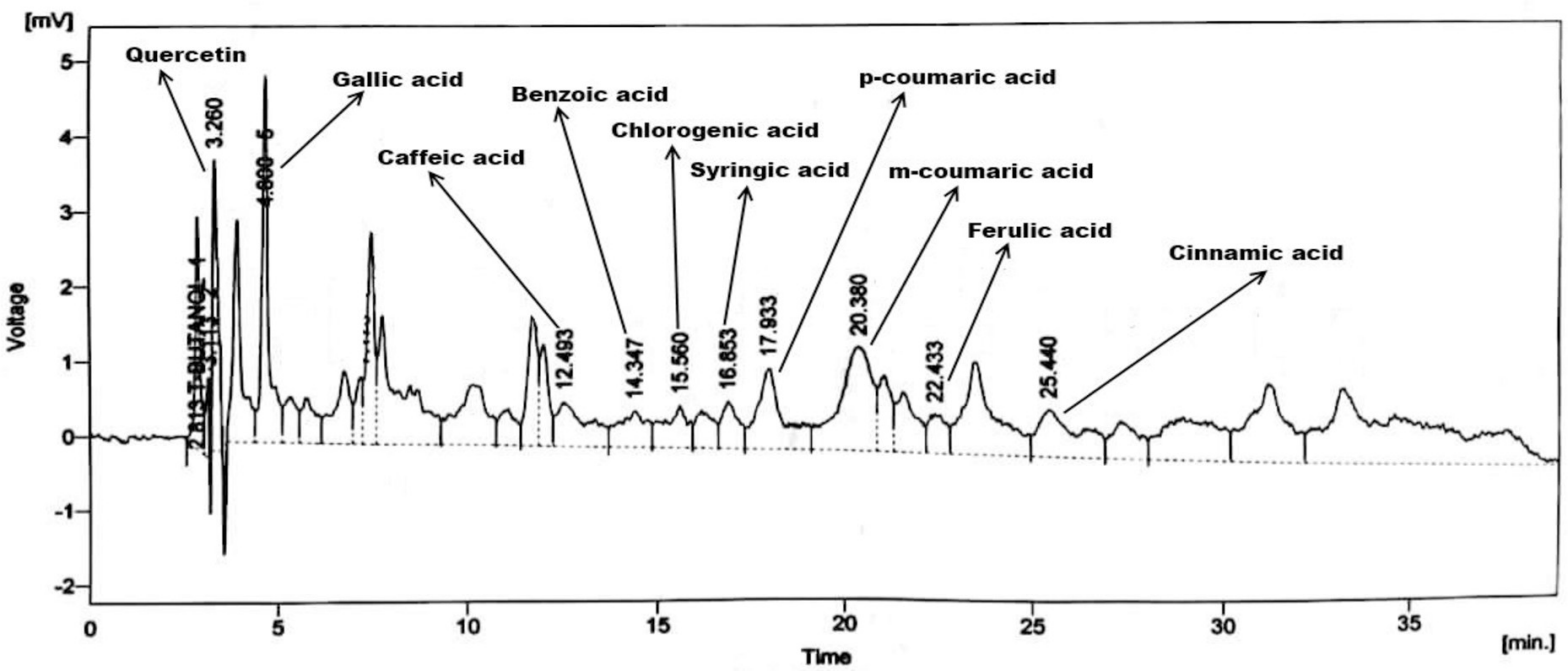
HPLC chromatogram of the *Euphorbia nivulia* Buch.-Ham. butanol extract.

The chromatographic fingerprinting and quantitative sketching of *E*. *nivulia* extracts were done by HPLC analysis (Figs 3–5). The results confirmed the presence of polyphenols include quercetin, gallic acid, caffeic acid, vanillic acid, benzoic acid, chlorogenic acid, syringic acid, and ferulic acid in *E*. *nivulia* crude, butanol, and aqueous extracts using the library of external standards of polyphenols for calculation of polyphenol quantities in the same extracts. The presence of various polyphenols, i.e., quercetin, gallic acid, caffeic acid, syringic acid, coumaric acid, ferulic acid, and cinnamic acid in *E*. *nivulia* crude extract has already been reported (Table 2) [32]. These polyphenols were recorded as 2.13, 2.19, 1.55, 2.59, 1.85, 0.47, 0.68, 1.58, 1.22 and 1.63 (ppm/mg) in the butanol extract (Table 3). While the same polyphenols were quantified in aqueous extract of *E*. *nivulia* as 1.87, 1.24, 0.81, 1.22, 1.57, 0.92, 0.32, 2.48 ppm/mg, respectively (Table 4).

**Table 2:** The qualitative and quantitative analysis of phenolic compounds in the *Euphorbia nivulia* Buch.-Ham. crude extract using HPLC.

Compounds	Retention time (min)	Area (mV s)	Phenolic content quantity (ppm/mg)
1. Quercitin	3.36	27.85	1.47
2. Gallic acid	4.58	27.59	0.99
3. Caffeic acid	12.85	9.55	0.43
4. Syringic acid	16.25	9.78	0.24
5. m-coumaric acid	20.14	16.67	0.19
6. Ferulic acid	22.15	17.43	1.25
7. Cinnamic acid	25.38	2.97	0.11

**Table 3:** The qualitative and quantitative analysis of phenolic compounds in the *Euphorbia nivulia* Buch.-Ham. butanol extract using HPLC.

Compounds	Retention time (min)	Area (mV s)	Phenolic content quantity (ppm/mg)
1. Quercitin	3.26	37.86	2.13
2. Gallic acid	4.60	61.19	2.19
3. Caffeic acid	12.49	33.71	1.55
4. Benzoic acid	14.34	24.51	2.59
5. Chlorogenic acid	15.56	23.83	1.85
6. Syringic acid	16.85	18.90	0.47
7. p-coumaric acid	17.93	52.81	0.68
8. m-coumaric acid	20.38	83.84	1.58
9. Ferulic acid	22.43	17.18	1.22
10. Cinnamic acid	25.44	46.69	1.63

**Table 4:** The qualitative and quantitative analysis of phenolic compounds in the *Euphorbia nivulia* Buch.-Ham. aqueous extract using HPLC.

Compounds	Retention time (min)	Area (mV s)	Phenolic content quantity (ppm/mg)
1. Quercitin	3.23	35.47	1.87
2. Gallic acid	4.48	34.60	1.24
3. Caffeic acid	12.20	17.74	0.81
4. Vanillic aid	13.58	19.62	1.22
5. Benzoic acid	14.70	14.97	1.57
6. Chlorogenic acid	15.32	11.82	0.92
7. Syringic acid	16.14	13.04	0.32
8. Ferulic acid	22.02	34.57	2.48

### 3.4. Phytotoxicity bioassay

Table 5 showed the phytotoxic effects of *E*. *nivulia* extracts against *L*. *minor*. All concentrations of hexane and water extracts showed no phytotoxic effects. Except for 100 μg/mL of the butanol extract, all other extracts at 10 and 100 μg/mL resulted in no effects on the growth regulation of *L*. *minor*. By contrast, crude and chloroform extract treatments at 1000 μg/mL have higher effects with 63% and 100% growth regulation using, respectively.

**Table 5:** *In vitro* phytotoxic effects of different *Euphorbia nivulia* Buch.-Ham. extracts against *Lemna minor* L.

Concentration (μg/mL)	Crude extract		Chloroform extract		Butanol extract		Hexane extract		Aqueous extract	
	No. of fronds	% Growth regulation	No. of fronds	% Growth regulation	No. of fronds	% Growth regulation	No. of fronds	% Growth regulation	No. of fronds	% Growth regulation
**0**	57	0	41	0	44	0	41	0	41	0
**10**	57	0	41	0	44	0	41	0	41	0
**100**	57	0	41	0	42	4.5	41	0	41	0
**1000**	21	63.15	00	100	32	27.1	41	0	41	0
**Result**	Good activity at highest concentration		Significant activity at highest concentration		Low activity at highest concentration		No activity		No activity	

Standard drug: Paraquat (Mc. Laughlin et al. 1991) under incubation condition as 28 ± 1 ^o^C.

### 3.5. Insecticidal bioassay

**Fig 6:**
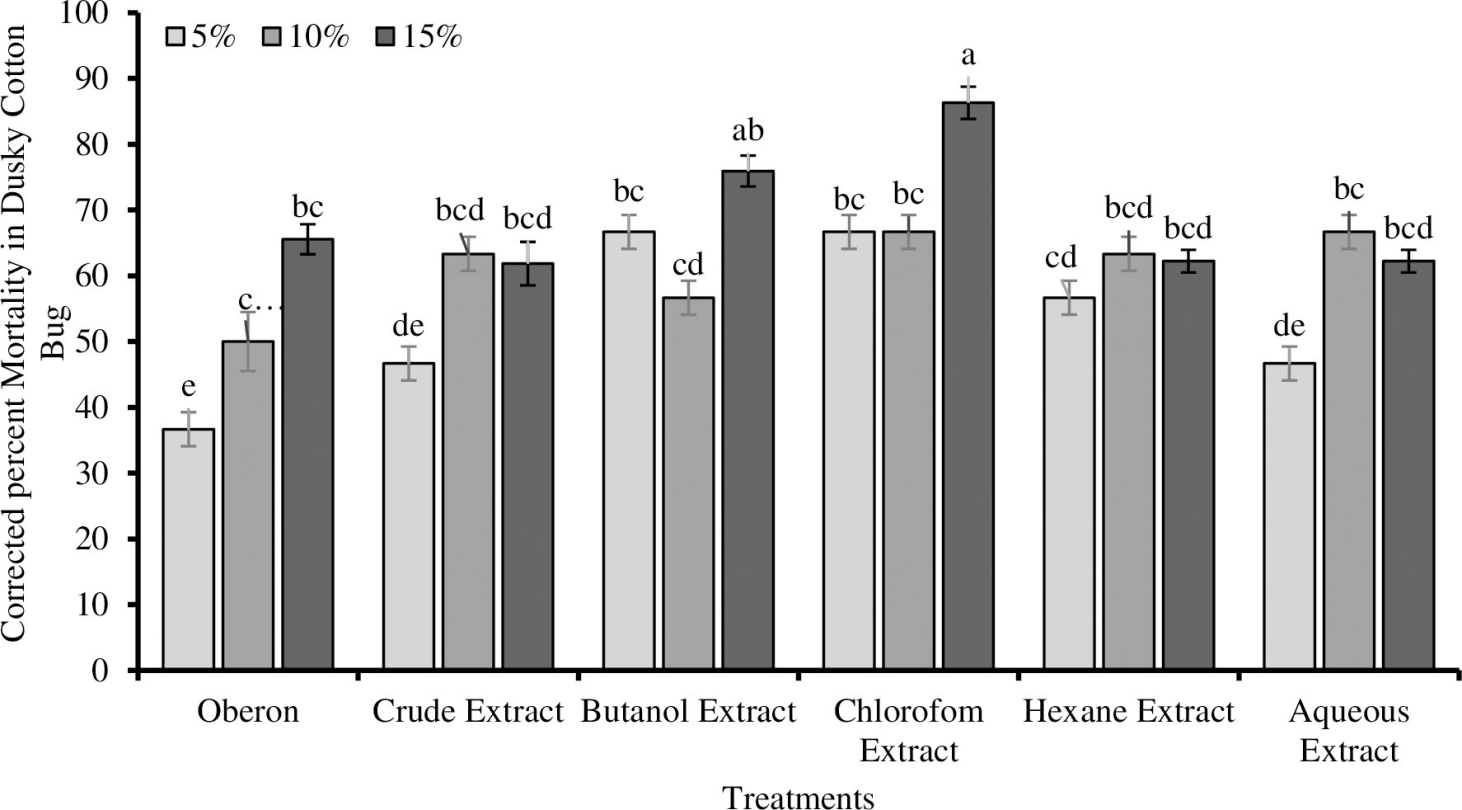
Corrected % mortality in *Oxycarenus hyalinipennis* (dusky cotton bug) after exposure to different solvent-based extracts of *Euphorbia nivulia* Buch.-Ham. and an insecticide Oberon, at different concentrations.

Fig 6 showed the effects of *E*. *nivulia* extracts on the mortality of *O*. *hyalinipennis*. It was found that the crude extract at 15, 10, and 5% concentrations resulted in 61, 62, and 48% mortality, respectively. Two of the highest mortalities were found as 87 and 75%, when *O*. *hyalinipennis* were treated with 15% concentrations of *E*. *nivulia* chloroform and butanol extracts, respectively. While, 15, 10, and 5% solutions of hexane and aqueous extracts showed 61, 62, 58, 61, 68, and 47% mortalities in *O*. *hyalinipennis*, respectively. The standard insecticide solution, Oberon, at concentrations of 15, 10, and 5%, resulted in 65, 50, and 36% mortalities in *O*. *hyalinipennis*, respectively.

## 4. Discussion

In *E*. *nivulia*, the investigation of phenolic compounds was limited to only a few compounds such as gallic acid, caffeic acid, and syringic acid [32]. In the present study, the phenolic profile of *E*. *nivulia* extracts was done by HPLC qualitative and quantitative analysis. It expanded the knowledge regarding various polyphenols, including quercetin, gallic acid, caffeic acid, vanillic acid, benzoic acid, chlorogenic acid, syringic acid, m-coumaric acid, p-coumaric acid, cinnamic acid, vanillic acid, and ferulic acid. The current research was further extended to investigate their phytotoxic and insecticidal potentials of crude extract and other solvent fractions of *E*. *nivulia*. It has been reported that phenolics, particularly water-soluble allelochemicals are responsible for the most allelopathic activities and thus, could cause growth-inhibitory effects to individual plants [33]. A few polyphenols, such as cinnamic acid derivatives (caffeic and p-coumaric acids), benzoic acid derivatives (vanillic and syringic acids), could impose deleterious impact (due to their water solubility) on their neighboring vicinity that present in adequate quantities [34].

Furthermore, phytochemicals, including phenolic acids, are germination and plant growth inhibitors [5]; this harmful effect of these phenolics might be due to their additive or synergistic actions [33]. Gibberellic acid controls α-amylase production and is also inhibited by phenolics [35]. These allelopathic phytotoxins affect the target species’ structural and physiological cell functions, leading to weakened germination and reduced growth [36].

Phytotoxic bioassay showed the depressive impact of *E*. *nivulia* crude and chloroform extracts on germination, growth, and some biochemical aspects of *L*. *minor*. The allelopathic potential of crude extract and its fractions in various concentrations was demonstrated in weeds’ germination. Significant (P ≤ 0.05) delay in the time to start germination and E50 over the control was provoked by the plant samples. The study’s findings confirmed the significant inhibitory effects against the tested species for seedling germination and growth. These significant phytotoxic effects are due to allelochemicals existing in the plant species affecting the different physiological processes, possibly through their effects on enzymes responsible for phytohormone synthesis and the inhibition of nutrients movement ion absorption by affecting plasma membrane penetrability [37].

Similarly, significant inhibition was observed at the higher concentrations of the plant extracts. The decreased plant germination may result from the allelochemical stress due to inhibition of water uptake [38] and altered activity of gibberellic acid, which is known for regulation of amylase production during the germination course. Cell elongation and division may also be inhibited by allelochemical [39]. The release of phytotoxins from incorporated crop residues by leaching or decomposition leads to reduced growth and development [40]. Allelopathic compounds are rapidly solubilized and imbibed by the germinating seeds, retard or delay emergence, and adversely affect the subsequent seedling growth. Phenolics, glycosides, terpenoids, and other secondary metabolites found in the crude extract and various plant fractions can exhibit phytotoxicity in plants [41–43]. Several workers have reported the allelopathic property of saponins [44,45] and amino acids against plants [20]. Phenolic compounds with their derivatives play an inhibitory role in stressing germination and seedling growth. They have allelopathic applications as herbicides in agriculture and forestry [34]. Phytotoxins may affect the membrane absorbency, ion-uptake, photosynthetic electron transport and respiratory chains, enzyme activity, and cell division [46]. Hence, it can be inferred that these active ingredients detected in this species may be responsible for *Lemna* plant growth inhibition.

*E*. *nivulia* extracts showed strong insecticidal activity because euphorbiaceous plants contain chemical constituents like triterpenoids and related compounds (alcohols, sterols, and hydrocarbons), phenolic compounds, alkaloids, cyanogenic glucosides, and glucosinolates [47]. Previously, several studies had shown pesticidal and insecticidal effects of *Euphorbia* plants [48]. Euphorbia plants are rich in latex, and latex toxicity is well established and reported. For example, the latex of *E*. *tirucalli* is composed of a range of toxic substances, including phenolics, ellagic acid and tannins [49], triterpenes [50,51], and diterpene esters [52]. These are the most relevant compounds to entomotoxicity and numerous biological activities [53,54]. Similarly, insecticidal properties of the latex of *E*. *antiquorum* have been reported previously De. Silva et al [16]. This is probably the first report on the phytotoxic and insecticidal potentials of *E*. *nivulia* crude extract and its various fractions. In our study, the mortality increased with increased concentration at all the doses at 96h exposure to the extracts showing remarkable insecticidal activity against the dusky cotton bug. Previously, *N*. *tobacum* and *C*. *procera* exhibited significant mortalities in *O*. *hyalinipennis* during various phytochemicals screening [24]. It is an indication to promote green chemistries for the effective management of agricultural pests. Indeed, phytochemical analyses and screening for active biological compounds can help greatly in this regard.

## 5. Conclusion

The findings of the present study suggested that chloroform and crude extracts of *E*. *nivulia* have good phytotoxic potential at their higher concentration. In contrast, all solvent-based extracts showed variable insecticidal potential against dusky cotton bugs. This might be due to different chemical constituents are extracted with different solvents with different actions. So, further studies of phytochemicals extracted with different solvents and conditions from *E*. *nivulia* could be focused on the selective action against harmful weeds and insect pests of field crops to avail opportunities to benefit sustainable agriculture in the future.
